# The Synergistic Effect of Limestone Powder and Rice Husk Ash on the Mechanical Properties of Cement-Based Materials

**DOI:** 10.3390/ma17205058

**Published:** 2024-10-16

**Authors:** Jialei Wang, Feifei Jiang, Juan Zhou, Zhongyang Mao

**Affiliations:** 1 School of Civil Engineering and Architecture, NingboTech University, Ningbo 315100, China; wangjl@nit.zju.edu.cn; 2 School of Civil Engineering, Nantong Institute of Technology, Nantong 226000, China; 3 College of Materials Science and Engineering, Nanjing Tech University, Nanjing 211800, China; mzy@njtech.edu.cn

**Keywords:** limestone powder, rice husk ash, synergistic effect, mechanical properties

## Abstract

Fully utilizing solid waste as supplementary cementitious materials (SCMs) while ensuring the mechanical properties of cement-based materials is one of the pathways for carbon reduction in the cement industry. Understanding the effects of the two solid wastes-limestone powder (LP) and rice husk ash (RHA) on the mechanical properties of cement-based materials is of great significance for their application in concrete. This study investigates the impact of LP and RHA on the strength of cement mortar at various ages and the microhardness of hardened cement paste. The results suggest that two materials have a certain synergistic effect on the mechanical properties of the cementitious materials. The addition of RHA effectively addresses the issues of slow strength development, insufficient late-stage strength of the cementitious material, and the low strength blended with a large amount of LP, while a suitable amount of LP can promote the strength increase in the cement-RHA system. Based on the comprehensive analysis of compressive strength and microhardness, the optimal solution for achieving high mechanical properties in composite cementitious materials is to use 10% each of LP and RHA, resulting in a 9.5% increase in 28 d strength compared to a pure cement system. The higher the content of LP, the greater the increase caused by 10% RHA in compressive strength of the composite system, which makes the strength growth rate of cementitious material mixed with 10% LP at 3–56 d 62.1%. When the LP content is 20% and 30%, the addition of 10% RHA increases the 28 d strength by 44.8% and 38.8%, respectively, with strength growth rates reaching 109.8% and 151.1% at 3–56 d.

## 1. Introduction

Cement production is one of the main sources of carbon and pollutant gas emissions, with carbon emissions from producing Portland cement estimated at around 0.7–0.9 t/t according to the statistics [1]. Carbon emissions generated by China’s cement industry in 2021 was approximately 1.375 billion tons, accounting for 13.5% of the country’s total emissions [2]. The reduction of clinker to cement ratio is crucial for decreasing the carbon emissions and achieving low-carbon production [3]. The resource utilization of solid waste is an effective way to reduce carbon emissions in cement production [4], using solid waste with potential pozzolanic activity as SCMs to replace cement. This approach aims to reduce the use of cement clinker from application aspects to production sources in order to control the carbon emissions [5]. However, it is essential to ensure that the main properties such as mechanics of the cement composite materials partially replaced by solid waste are not significantly affected, which raises higher requirements for the selection of solid waste.

Limestone is one of the raw materials for cement production and the major source of carbon emissions from clinker. As a by-product of limestone minerals, LP has been widely studied as a mineral admixture to replace cement. Although it reduces cement usage from an application perspective, the addition of LP has an adverse impact on the mechanical properties of cement-based materials and their development with age, which has also led to a significant reduction in its utilization rate. The nucleation effect of LP particles promotes the deposition of hydration products, which can accelerate the hydration of cement particles to improve the early strength of cement paste [6]. However, LP does not have pozzolanic activity and the hydration reaction with C_3_A in cement clinker is relatively slow. The reduction in cement content caused by dilution effect decreases the amount of hydration products, and excessive amounts of LP negatively impact the late-stage strength of cement-based materials and its development with age [7]. Specifically, adding more than 10% LP can adversely affect the compressive strength of cement paste [8], while exceeding 20% leads to a reduction in strength with the increasing LP content [9]. It is well known that the pore structure is one of the factors that affect the strength of cement-based materials, but the dilution and dissolution behavior of LP can cause the coarsening effect of pores with age [10], which is also the reason for insufficient development of the strength. In order to address the issue and promote the application of LP in cement-based materials, a more reasonable approach is to use other solid-waste materials for system improvement and optimization.

As a type of agricultural solid waste, RHA contains a large amount of active SiO_2_ under certain conditions, making it an ideal SCM that seems to be able to mitigate the adverse effects of LP. RHA has the characteristics of high specific surface area and porous structure, which has impacts on the strength of cement paste in various aspects [11,12]. RHA can provide nucleation sites for cement hydration, promote the hydration reaction of cement, and the fine particles can fill the pores in the paste. The pozzolanic reaction consumes Ca(OH)_2_ (CH) and forms C-S-H gel to further fill the pores and enhance the density of pastes. Additionally, the porous structure facilitates moisture release to promote the further hydration of cement. As a result, the compressive strength and its development of cement-based materials is significantly improved under the physicochemical action of RHA particles [13], along with corresponding increases in tensile and flexural strength [14,15,16].

The application of LP and RHA in cement-based materials has been studied. Sensale [11] found that the 28 d compressive strength of the 5% LP samples blended with 10%, 15%, 20%, 25%, and 30% RHA, respectively, was the highest compared to other mixtures blended with LP. Sua-iam [17] showed that self-compacting concrete prepared with LP and RHA exhibited higher mechanical properties than the ordinary self-compacting concrete, reaching a strength of 40 MPa at 28 d, an increase of 13%. In a quaternary composite cementitious system, the compressive and flexural strength of 20% fly ash mixed with 10% LP and 10% RHA to replace cement have been improved to varying degrees [18].

However, the application composite system mixed with LP and RHA still has research gaps, and the impact of their synergistic effect on the mechanical properties of the materials has not been clearly expressed. Particularly, the research on their mechanical properties also mainly focuses on the analysis of strength data, lacking studies on the micromechanical properties of the composite system. The role of RHA in enhancing the mechanical properties of composites with high LP content remains unclear, which is crucial for the feasibility of using the composite approach to improve the utilization rate of LP. Therefore, this study aims to improve the low strength and its slow development of the cement-LP system by utilizing the high pozzolanic activity and unique porous structure of RHA. The influence of the interaction between LP and RHA on the mechanical properties of the composite cementitious system was investigated through the strength of mortar and the microhardness of hardened cement paste. The research provides a relevant basis for the feasibility application of using LP and RHA as composite replacements for cement, which can not only improve the comprehensive utilization rate of these two types of solid waste in engineering materials, but can also effectively reduce the high carbon emissions caused by cement application to a certain extent. Therefore, the application of LP and RHA in cementitious materials is of significant importance in engineering.

## 2. Experimental

### 2.1. Materials and Mix Proportion

**Figure 1:**
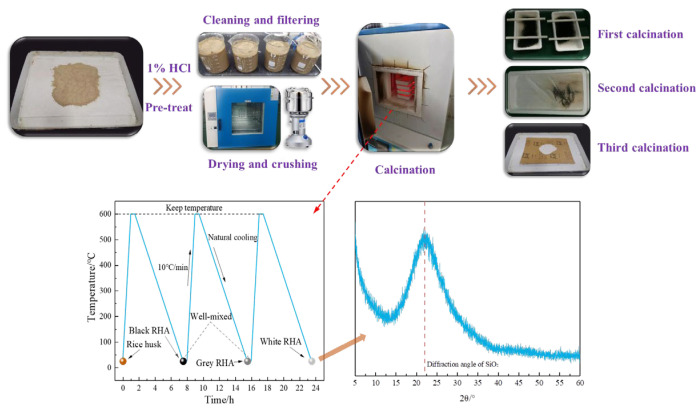
Combustion program and preparation process of RHA.

**Figure 2:**
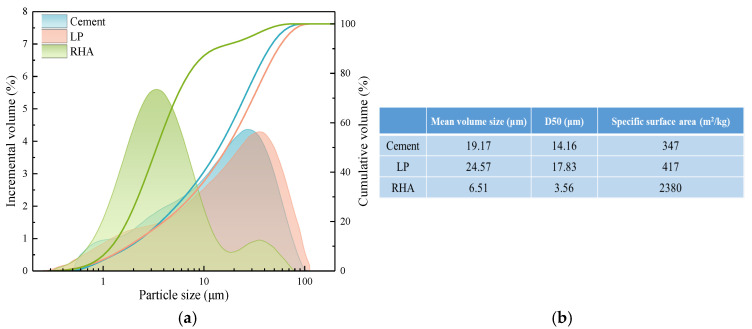
The (**a**) particle size distribution and (**b**) particle parameters of cement, LP, and RHA.

P·I 42.5 Portland cement produced by China United Cement Group Co., Ltd. was used to prepare mortar and paste, with a density of 3.14 g/cm^3^, which was marked as P. The LP (produced in Jingmen, China) contained 98.22% CaCO_3_ with a density of 2.7 g/cm^3^, which was marked as L. The preparation of RHA [19,20]: grinding rice husk into powder and soaking it in a 1% dilute hydrochloric acid solution for 1 h to fully acidify the rice husk powder. After pretreatment, the powder was washed and filtered 3 times with clean water, oven-dried, and then heated in a muffle furnace to obtain white RHA, with the combustion program (the calcination needs to be carried out in 3 times) and preparation process shown in Figure 1. After homogenization, the density of RHA was measured at 2.07 g/cm^3^, marked as R. The particle size distribution and particle parameters of the powder materials are shown in Figure 2, and the chemical composition of the cement and RHA is listed in Table 1.

The fine aggregates were made of quartz sand with a fineness modulus of 2.8, categorized as medium sand in Zone II, with continuous grading. The Sika-3301H type polycarboxylic superplasticizer was adopted, which was diluted from a stock concentration of 55% to 25% before test. The tap water was used for mixing.

**Figure 3:**
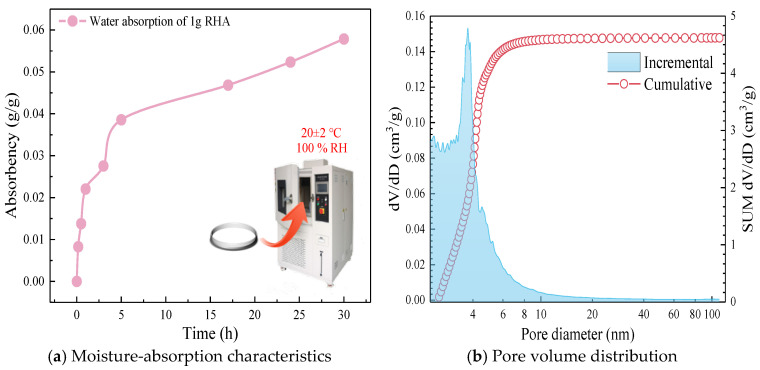
Characterization of pore structure characteristics of RHA.

**Figure 4:**
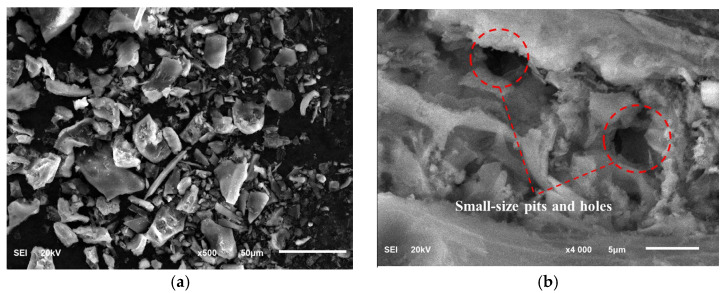
SEM images (**a**) ×500 and (**b**) ×4000 of RHA.

According to Table 1 and Figure 1, the main component of RHA is amorphous SiO_2_. The amorphous SiO_2_ has high reactivity and can rapidly participate in secondary hydration reaction within cement composite systems, consuming calcium hydroxide and generating more C-S-H gel, which can improve the strength of cement-based materials to some extent. The combustion of organic matter results in a porous powder [21], and its porous structure provides more internal surfaces, which makes the specific surface area (SSA) of RHA much larger than that of cement and LP. In order to confirm the porous structure characteristics of RHA, the hygroscopic properties were tested [22,23]. A certain amount of RHA was spread in a glass petri dish and placed in a constant temperature and humidity chamber to monitor changes in sample mass with time, as shown in Figure 3a. The N_2_ adsorption-BJH method was used to obtain the adsorption isotherm of pore volume distribution, with the pore volume distribution illustrated in Figure 3b. Based on the cumulative pore volume per unit mass, the porosity of RHA was 67.04%. Figure 4 shows the microscopic morphological characteristics, which reveals that the particles are polygonal and angular, with pore structures primarily consisting of small pits and holes, exhibiting certain water storage capability. The experimental mix proportions are detailed in Table 2.

### 2.2. Test Methods

#### 2.2.1. Raw Material Performance Tests

##### Particle Size Distribution (PSD)

The PSD test was conducted using a Malvern-Mastersizer 2000 type laser particle size analyzer produced in Malvern, PA, USA. The refractive index of solid particles was set at 0, and the refractive index of the dispersant was set at 1.33, with opacity ranging from 4% to 8%. The testing range was from 0.02 to 2000 μm. Ethanol was used as the dispersant for the testing of cement, LP, and RHA.

##### X-Ray Fluorescence (XRF)

XRF analysis was used to determine the types and content of elements in the powder. The sample to be tested was ground using an agate mortar, and an Axios mAX type XRF spectrometer produced by PANalytical B.V. in Almelo, The Netherlands was employed for testing, yielding the elemental mass ratio or the mass ratio of oxides in the material.

##### N_2_ Adsorption Test

Dynamic chromatography was used to measure the nitrogen gas adsorption capacity. Based on the nitrogen isothermal adsorption or desorption curves, the SSA of RHA was obtained. Before conducting the adsorption measurement, the physical adsorbates on the surface of the adsorbent should be removed through “degassing”. The RHA powder to be tested was first placed in a U-shaped sample tube, allowing a mixed gas containing a certain proportion of adsorbate to flow through the sample. The adsorption amount of the sample for the adsorbate molecules (N_2_) was determined by measuring the change in gas concentration before and after adsorption. By measuring the amount of nitrogen adsorbed by the sample at a nitrogen partial pressure, the nitrogen isothermal adsorption or desorption curve can be plotted, thereby obtaining the SSA or pore size distribution of RHA.

##### Scanning Electron Microscope (SEM)

A JSM-6700F type SEM produced by JEOL in Tokyo, Japan was used to observe the surface morphology and pore characteristics of RHA powder particles. To prevent the powder from being removed under high vacuum and to enhance the conductivity of the powder particles, gold sputtering treatment was applied to the RHA. The vacuum condition was set to 5 × 10^−5^ Pa, and the operating voltage was 20 kV.

#### 2.2.2. Strength Test

**Figure 5:**
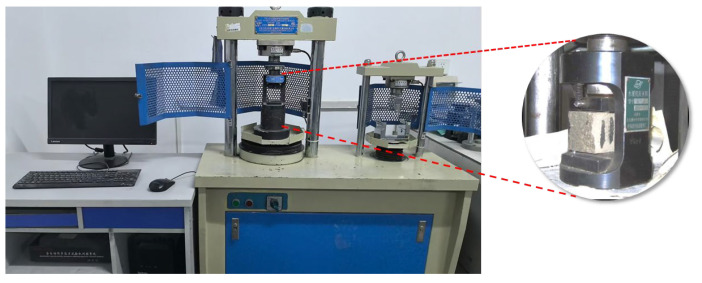
Compressive and flexural strength tests of cement mortar.

According to the mix proportions in Table 2, specimens sized 40 mm × 40 mm × 160 mm were prepared. The flexural and compressive strength of the corresponding mortar specimens were tested according to GB/T 17671-2021 [24] “Test Method of Cement Mortar Strength”. A DYE-300S flexural and compressive testing machine was used for the tests, with a loading rate of 2.4 kN/s, as shown in Figure 5.

#### 2.2.3. Microhardness Test

**Figure 6:**
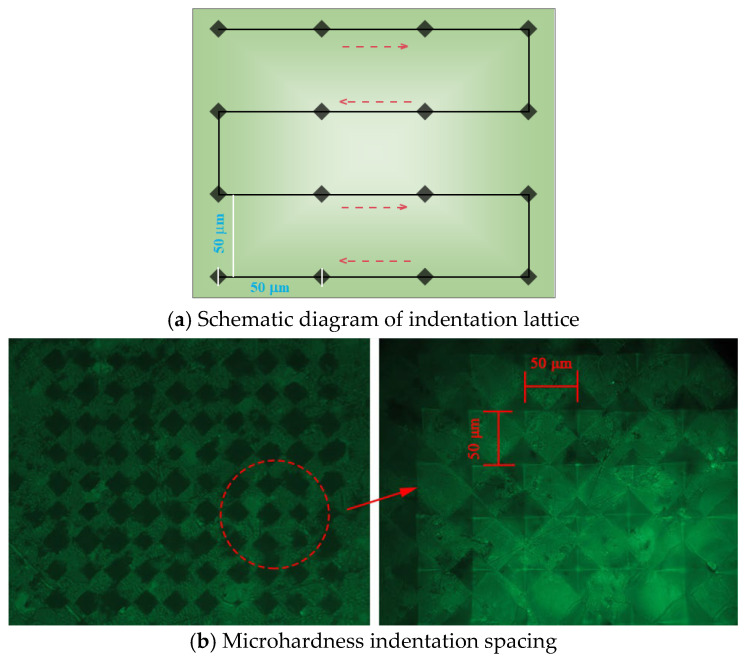
Indentation lattice of microhardness.

Based on the mix proportions in Table 2, an equal-proportional cement pastes without sand were prepared. The hardened samples at 28 d were cut into 2 mm thick slices and then broken into block samples of approximately 1–3 cm. Isopropanol was used to terminate hydration for 7 d, after which the samples were vacuum-dried. The dried samples were embedded in epoxy resin, and the epoxy on the testing surfaces was initially pre-ground with 400# sandpaper until samples nearly exposed. This was followed by further grinding with 600# sandpaper until the sample surfaces were fully revealed. Finally, the surfaces were polished to a smooth and reflective state by using 1200# sandpaper. The ground samples were then placed on a polishing machine and polished sequentially with 2.5 μm, 1 μm and 0.5 μm particle sizes of diamond polishing agents. The polished samples were tested using the HMAS-D1000M Vickers microhardness testing system produced by Shanghai Yanrun Optics Technology Co., Ltd., Shanghai, China, applying a load of 0.245 N for 15 s. A 10 × 10 serpentine dot matrix was used for indentation test, with the spacing between each pair of indentations adjusted to 50 μm according to the actual area of the indentation, as shown in Figure 6. The Vickers microhardness values were calculated according to Equation (1).

(1) $$HV=0.1891\times \frac{F}{d^{2}}$$

where *HV* is the microhardness value of a single indentation, MPa; *F* is the applied load, and all indentations are uniformly 0.245 N; *d* is the arithmetic average value of the diagonal of a single indentation, mm.

## 3. Results and Discussion

### 3.1. Strength of Composite Cementitious Materials

#### 3.1.1. Improvement of RHA on the Strength of Cement-LP System

**Figure 7:**
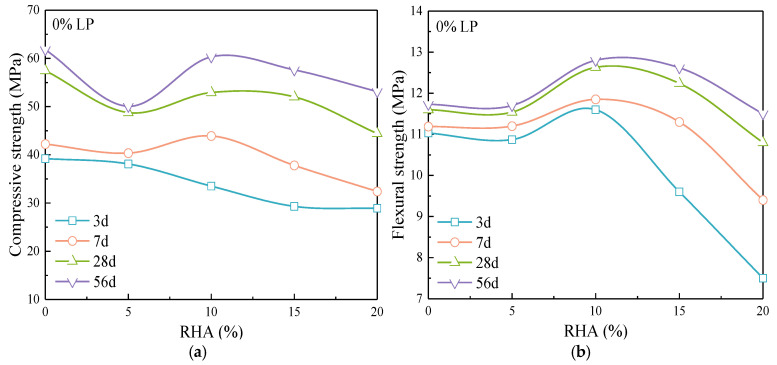
Effect of RHA on (**a**) compressive strength and (**b**) flexural strength of cement mortar.

Figure 7 shows the variation of compressive and flexural strength of cement mortar with the content of RHA. The effect of RHA on the compressive and flexural strength varies with different hydration ages. As shown in Figure 7a, the compressive strength of cement mortar decreases with RHA at 3 d age. When the age is 7 d, 28 d, and 56 d, 10% RHA significantly improves the compressive strength compared to the pure cement mortar. For 10% and 20% RHA content, the increasing rate in compressive strength accelerates with age, while for 5% RHA, the increase is relatively slow. Figure 7b indicates that 10% RHA also significantly enhances the flexural strength of cement mortar, with a higher content of RHA leading to greater increases with age.

The addition of the fine particles increases the hydration rate of cement [25]. The average particle size of RHA is much smaller than that of cement and LP, and the pores within the particles provide a larger SSA. RHA can offer the nucleation sites for cement hydration, and the embedded fine particles in the hydration products can better fill the pores of paste. The pozzolanic reaction consumes CH and forms new C-S-H gel, which continues to fill the pores. The porous structure promotes the release of moisture, enhancing the hydration of both cement and RHA, and further improving the density of the mortar [26]. High content of RHA can severely limit the water requirement for early-age cement hydration, significantly reducing the hydration degree. After the paste hardens, the RHA gradually releases water as the humidity changes, which facilitates the formation of hydration products and fills the pores, thus increases the density. The higher the RHA content, the greater the amount of water released, which more significantly promotes the hydration, resulting in a relatively larger increase in the strength with age.

**Figure 8:**
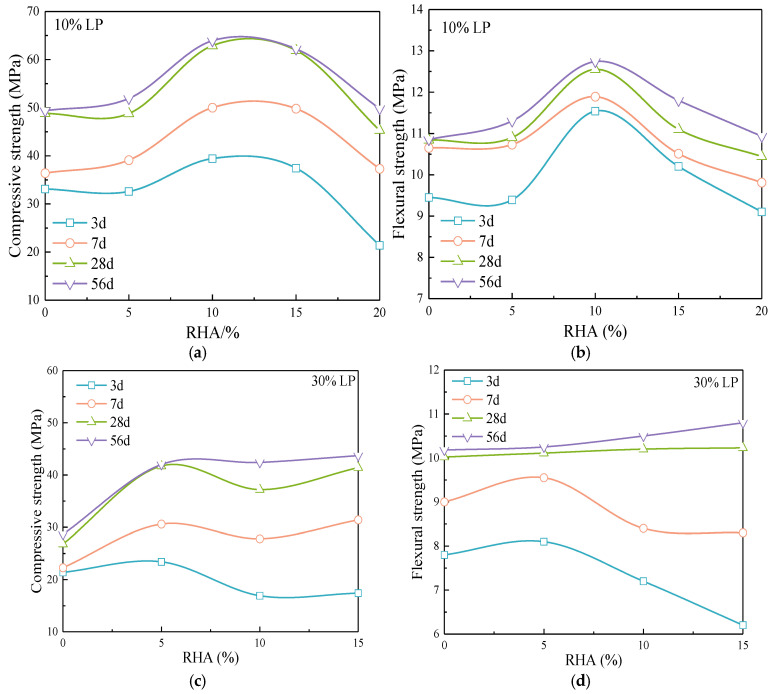
Effect of RHA on (**a**,**c**) compressive strength and (**b**,**d**) flexural strength of cement-LP composite mortar.

Figure 8 shows the effect of RHA on the strength of cement-LP mortar. The compressive and flexural strengths of the mortar blended with LP initially increase and then decrease with the content of RHA. The highest mortar strength is achieved with a combination of 10% LP and 10% RHA, resulting in a 9.5% increase in strength at 28 d compared to pure cement mortar. When the age is 7 d, 28 d, and 56 d, 5%, 10% and 15% RHA significantly enhance the strength of the mortar mixed with 10% LP, and 20% RHA increases the late-stage strength growth of the composite mortar, which makes the strength at 28 d and 56 d closer to that of the mortar with 10% LP.

Figure 8c,d compare the effect of RHA on the mortar strength when the LP content is 30%. Five percent RHA improves the compressive strength at 3 d age. With the age increases, the higher content of RHA leads to a significant improvement in compressive strength. The higher the content, the more water is released in the late stage, and the more significant the promoting effect on hydration, thereby facilitating the rapid development of later strength.

The flexural strength of the composite mortar mixed with 30% LP exhibits different trends at various ages. The strength initially increases and then decreases with the RHA content at 3 d and 7 d, and it increases with the content at 28 d and 56 d, which indicates that the increased RHA enhances the growth rate of flexural strength with age in the mortar blended with 30% LP. For mortar with a high LP content, 5% RHA effectively improves the strength at 3 d and 7 d, while 10% and 15% RHA enhance the growth rate of strength with age.

**Figure 9:**
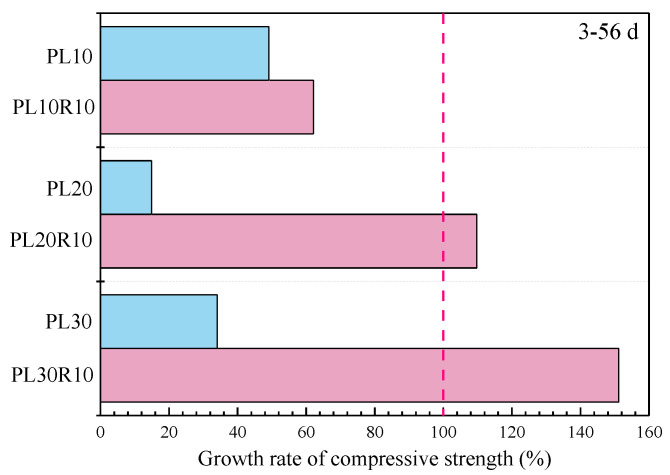
Effect of RHA on compressive strength growth rate of cement-LP mortar.

Figure 9 shows the effect of 10% RHA on the compressive strength growth rate of composite systems with different LP contents at 3 d–56 d. The addition of RHA results in higher compressive strength growth rates for the composite mortar compared to the cement-LP system. The improvement in compressive strength is greater with the higher LP content; the growth rate is 62.1% for PL10R10, while PL20R10 and PL30R10 reach growth rates of 109.8% and 151.1%, respectively, indicating a significant increase in compressive strength. This comparative analysis shows that the addition of RHA effectively addresses the issues of slow strength development and insufficient late-stage strength in high LP content systems.

#### 3.1.2. Effect of LP on Strength of Cement-RHA System

**Figure 10:**
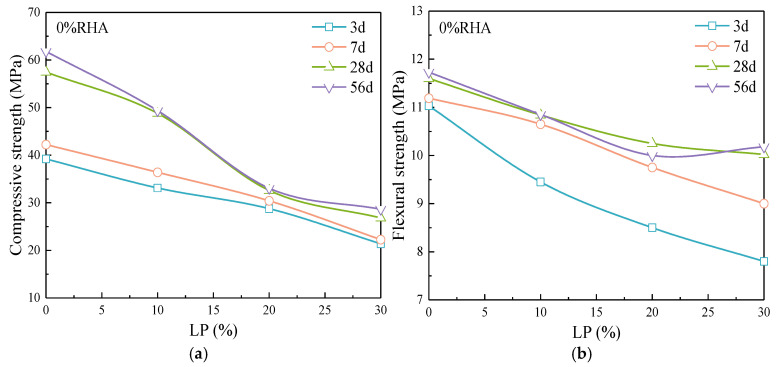
Effect of LP on (**a**) compressive strength and (**b**) flexural strength of cement mortar.

Figure 10 illustrates the changes in compressive strength and flexural strength of cement mortar with varying LP content. The compressive strength of the cement mortar decreases with LP content at all the ages. In the early stages of hydration, the addition of a small amount of LP does not enhance the strength of the cement paste, which differs from the previous studies [27,28]. This is largely due to the significant influence of LP particle size on early strength [29], which is related to the average particle size of LP [30].

**Figure 11:**
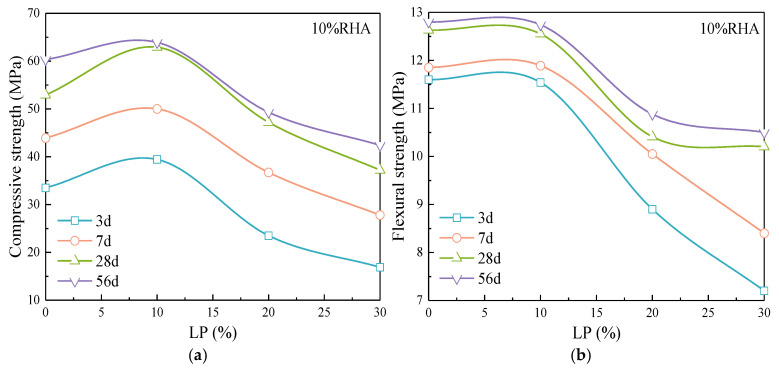
Effect of LP on (**a**) compressive strength and (**b**) flexural strength of cement-RHA mortar.

During the initial mixing and plastic state of the cement paste, the nucleation effect of LP allows for effective dispersion and enough hydration of the cement particles. The LP particles embedded in the hydration products also serve as good fillers, forming a favorable particle packing effect that leads to rapid early strength growth of the mortar with the age. However, the average particle size of the LP is 24.57 μm (Figure 2b), which is coarser than that of the cement particles, resulting in a relatively weaker acceleration effect on cement hydration. Thereby, it leads to a decrease in early (3d, 7d) strength of the mortar as the LP content increases. When the LP content is 20% and 30%, the development of strength in the late stage (>7d) is slow, with the strength increase at 7 d–28 d significantly lower compared to the 10% content. Insufficient late-stage strength growth is the major issue in the cement-LP system. The trend of flexural strength variation with LP content is similar to that of the compressive strength, showing rapid growth in the early stage and slow growth in the late stage. However, the flexural strength at 56 d blended with 20% and 30% LP shows an anomalous trend. This is due to the relatively low flexural strength values of the cement mortar, and the higher content of LP leads to a significant reduction in later-stage flexural strength. As a result, certain numerical errors are inevitably present in the lower strength values. Figure 11 shows the effect of LP on the strength of the cement-RHA composite mortar.

Due to the extremely low reactivity of LP, the hydration degree of the cementitious materials is significantly reduced. As a result, the strength of mortar blended with a high content of LP is markedly reduced at all the ages. The interaction between LP and RHA has a certain synergistic effect on the hydration of cement. The nucleation effect of RHA can influence the nucleation of hydration products to some extent, and the incorporation of LP can effectively alleviate the issue of insufficient nucleation sites for cement particles. Due to the intense pozzolanic reaction of RHA and the accompanied hydration of cement at an early stage, an appropriate amount of LP mitigates the competitive relationship for nucleation sites between the hydration products of cement and RHA. Both of them can effectively undergo the hydration reactions, which can increase the content of the products and thereby enhances the strength of the mortar. In summary, the cement-LP system exhibits slow strength growth in the late-stages, and excessive LP content can lead to a significant reduction in the strength at all the ages.

#### 3.1.3. Synergistic Effect of LP and RHA

**Figure 12:**
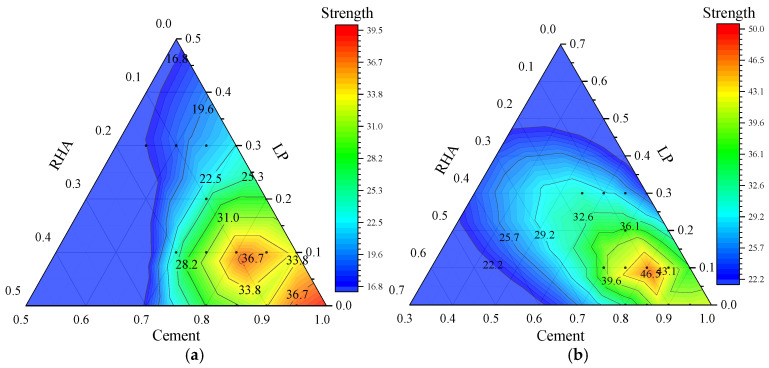

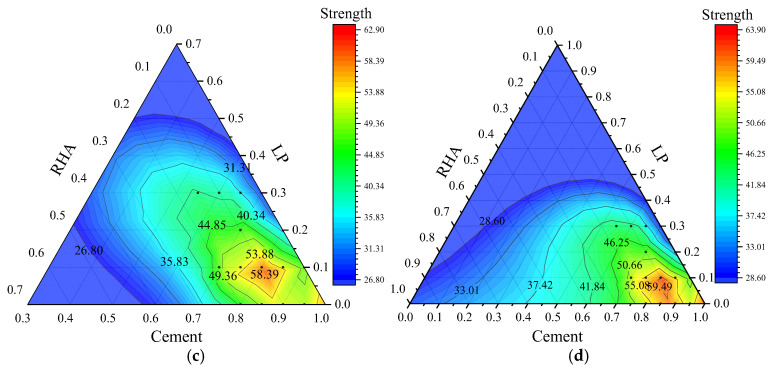
Effect of LP and RHA on compressive strength (**a**) 3 d, (**b**) 7 d, (**c**) 28 d, and (**d**) 56 d in composite system.

Figure 12 presents the analysis of the synergistic effects of LP and RHA on the compressive strength of the composite cementitious system. The overall trend in compressive strength at various ages indicates a decrease with the LP content and an increase with the RHA content. For the compressive strength of the composite mortar at 3 d and 56 d, the primary influence factors are the contents of LP and RHA. However, the compressive strength at 7 d and 28 d is greatly affected by the LP content, with strength gradually decreasing as LP content increases.

At the age of 3 d, 7 d, and 28 d, a peak strength region is observed when the LP content is at 10–20% and the RHA content is at 5–15%. There is an additional peak strength region at 3 d age when both LP and RHA contents are in the range of 0% to 10%. At the age of 56 d, a peak strength region is also observed when the LP content is at 10–20% and the RHA content is at 5–20%. This indicates that an appropriate increase of RHA content significantly enhances the compressive strength of the cement-LP system in the late-stage.

### 3.2. Micromechanical Properties

**Figure 13:**
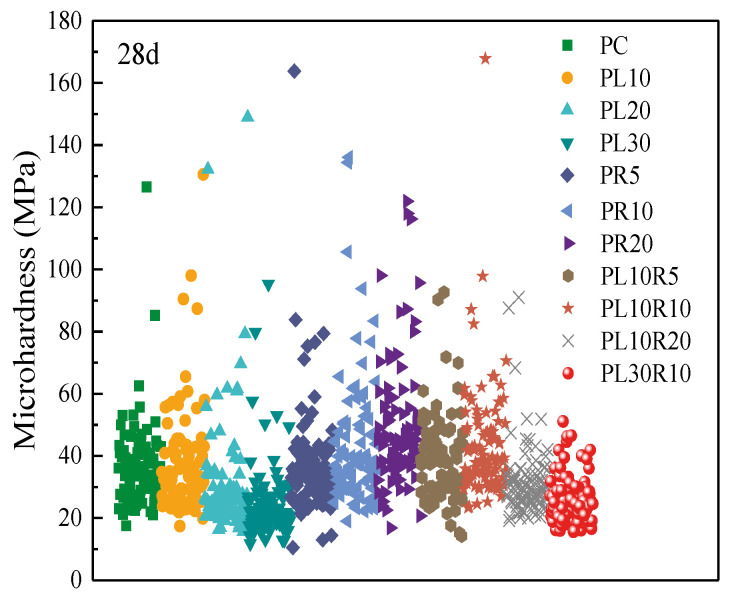
Microhardness of cement-LP-RHA composite paste.

The macro-mechanical properties of cement-based materials are closely related to the microstructure. In addition to the pore structure, the load-bearing capacity of hardened cement paste under stress is crucial, which is related to the hardness of the cement paste. Microhardness effectively characterizes the ability of the cement paste to resist the surface-embedding of hard objects. The microhardness of cement stone depends on the composition and density of the hydration products in the cementitious system. The incorporation of SCMs can change the amount and density of the cement hydration products, thereby affecting the hardness of the cement paste [31,32]. The hydration products of the cementitious system exhibit elastic-plastic characteristics under stress, and their non-uniform distribution at the microscale results in complex and unstable deformation under microstresses, leading to significant data dispersion in microhardness measurements. Therefore, mathematical statistical analysis of the microhardness data is necessary to reduce the substantial errors caused by this dispersion [33]. Due to the high variability in the microhardness test values of the cement paste, some values greater than 200 MPa were excluded. The microhardness testing results of the cement-LP-RHA paste at 28 d of hydration are shown in Figure 13.

Under the same load, the indentation area at different points varies, reflecting the differences in microhardness. This is primarily due to the differing physical properties of the hydration product at various indentation locations. Although there is significant dispersion in the microhardness test values of the cement paste, the microhardness at the indentation points mainly falls within the range of 10 MPa to 60 MPa. There are fewer hydration products with lower microhardness, and there are fewer unhydrated cement particles with higher hardness values, which indicates that the cement paste has a high degree of hydration.

**Figure 14:**
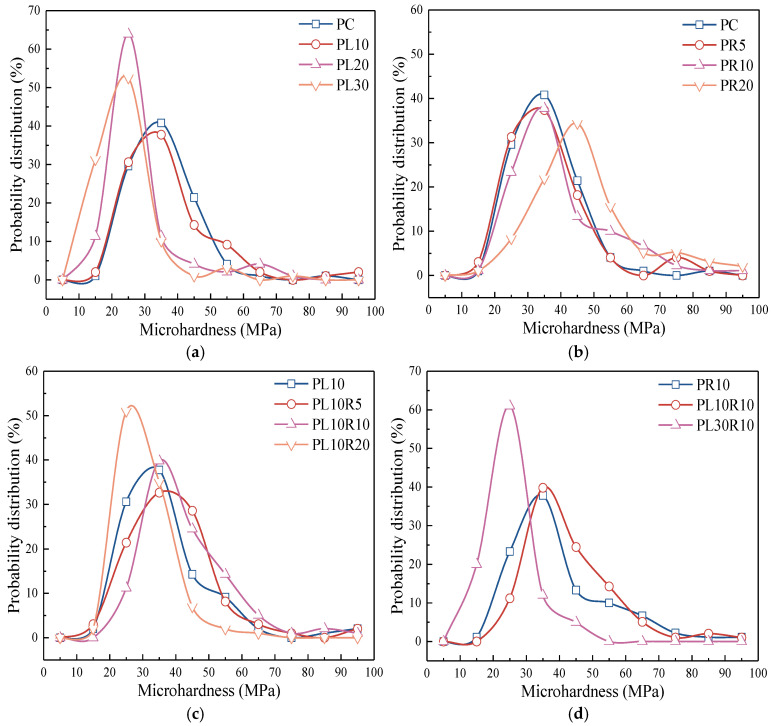
Probability distribution of composite paste microhardness in (**a**) cement-LP, (**b**) cement-RHA and (**c**,**d**) cement-LP-RHA system.

Figure 14 shows the probability distribution of microhardness. The overall trend in the probability distribution indicates that an increase in LP content reduces the hardness of the cement paste, as shown in Figure 14a,d. Particularly when the content exceeds 10%, the reduction becomes more pronounced. The addition of LP leads to a reduction in cement hydration productsand a looser bridging among the products and the formation of low-density C-S-H gel, which decreases the microhardness of the matrix. An appropriate content of RHA can effectively improve the microhardness, as shown in Figure 14b,c. The pozzolanic reaction of the RHA and its internal curing effect promote the cement hydration, leading to a denser interlayer structure of C-S-H and the formation of high-density C-S-H gel, thereby increasing the microhardness. When the RHA content is too high, it can lead to insufficient tightness in the connections between products, thereby affecting the hardness of the matrix.

**Figure 15:**
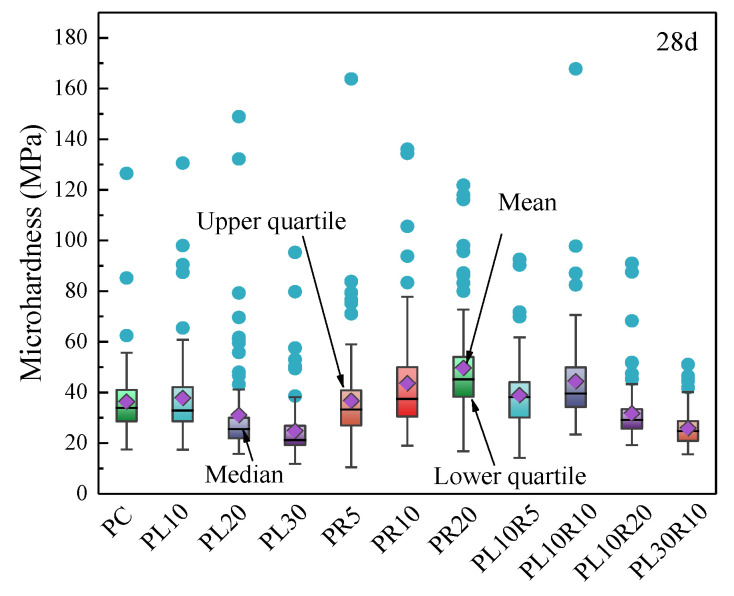
Box-plot of microhardness distribution of hardened cement paste.

The microhardness at various points within the indentation area shows a concentrated distribution range along with some discrete data points. Based on the theoretical and statistical perspective, when there are enough testing points in the indentation area (100 points in this test), the results should conform to a certain statistical laws. Box plots can display the distribution of the data and identify any outliers, as shown in Figure 15, with statistical parameters listed in Table 3. From the data distribution, the upper and lower quartiles of microhardness values for all the samples range from 18.2 MPa to 58.5 MPa, indicating a wide distribution with many discrete points. The significant dispersion in microhardness test values is primarily due to the heterogeneity of the cement paste, where the distribution of internal hydration product phases is random and uneven. Additionally, the sample preparation process can create certain defects on the indentation surface during grinding. When the diamond indenter of the microhardness tester presses on these defect areas during the pressing process, the defects are flattened first to make them even with the surrounding surface before they can be stabilized. Under the same load, the presence of a certain number of defects and irregular depth distribution leads to a relatively scattered measured microhardness value. However, as shown in the overall data distribution, the microhardness values tend to decrease with the LP content, while they increase with the RHA content.

**Figure 16:**
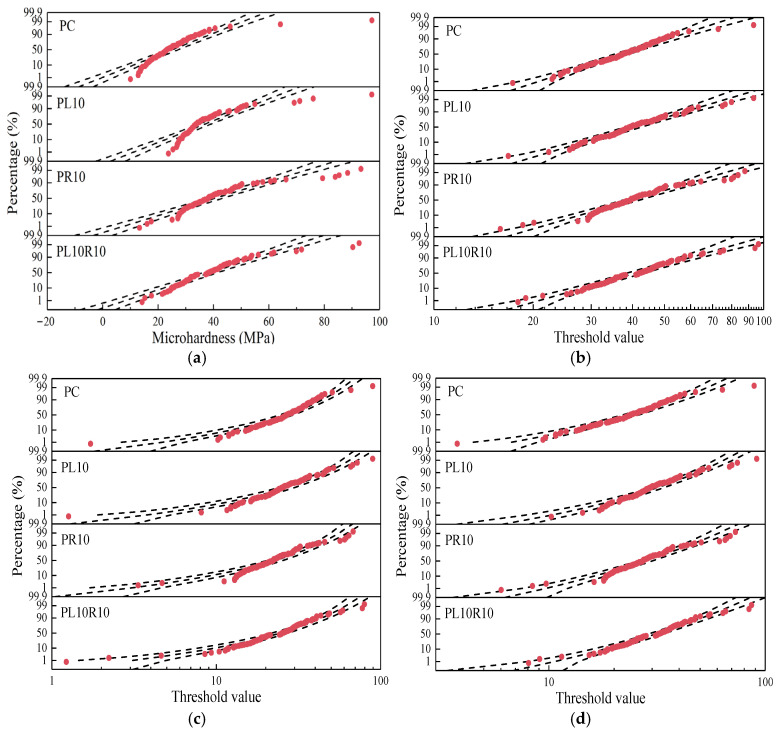
Fitting of microhardness data with different distributions (**a**) normal, (**b**) 3-Parameter Lognormal, (**c**) 3-Parameter Weibull, and (**d**) 3-Parameter Gamma of samples.

From the statistical parameters in Table 3, it can be seen that the microhardness of hardened cement paste does not simply follow a normal distribution [34]. The statistical distribution of the microhardness data was examined using Minitab 19.1 software, with the normal, 3-Parameter Lognormal, 3-Parameter Weibull, and 3-Parameter Gamma distributions shown in Figure 16.

In each distribution, the three lines represent the −95%, 100%, and 95% confidence intervals. If data follow a distribution, more points should fall within the −95% to 95% confidence interval region. From Figure 16, it is evident that the microhardness values of the samples are more consistent with the 3-Parameter Lognormal distribution. Minitab provides the statistical value representing the Anderson–Darling statistic, and typically the smaller the AD value, the more likely the data are to follow this distribution. The AD values are shown in Table 4, which further illustrates that the microhardness of the hardened cement paste conforms to the 3-Parameter Lognormal distribution rather than the normal distribution. This result is consistent with previous research findings [35,36].

Using the goodness of fit test in Minitab, the three parameter values corresponding to the 3-Parameter Lognormal distribution of the sample’s microhardness can be obtained: the threshold parameter *θ*, the location parameter *μ*, and the scale parameter *σ*. According to the statistical principles of the lognormal distribution, deconvolution statistical analysis can be employed to derive the probability density function (PDF) and cumulative distribution function (CDF) of the sample hardness values [37], as shown in Equations (2) and (3).

(2) $$f(u)=\frac{1}{\sigma \sqrt{2\pi }}\exp\left[-\frac{{\left(u-\mu \right)}^{2}}{2\sigma^{2}}\right]$$

(3) $$F(u)=\frac{1}{\sigma \sqrt{2\pi }}\int_{-\infty }^{u}\exp\left[-\frac{{\left(u-\mu \right)}^{2}}{2\sigma^{2}}\right]du$$

where *f*(*u*) represents the PDF of the sample’s microhardness values; *F*(*u*) is the CDF; *μ* is the mean of all the test values; *σ* is the standard deviation of the test values; and *u* is the microhardness value of a single test point in the sample.

**Figure 17:**
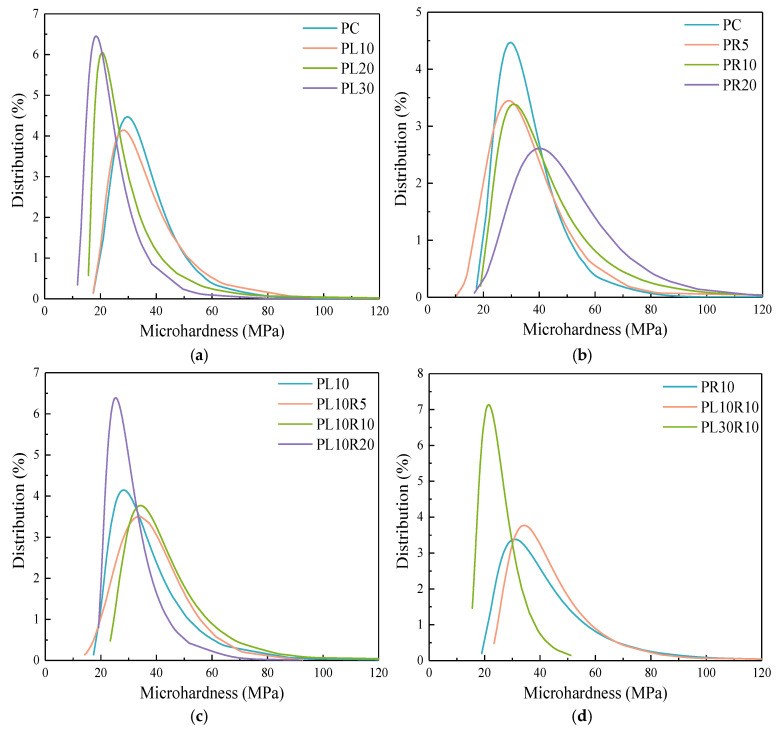
Probability density of 3-Parameter Lognormal distribution of microhardness data in (**a**) cement-LP, (**b**) cement-RHA and (**c**,**d**) cement-LP-RHA system.

Figure 17 shows the probability density curve of the lognormal distribution of microhardness. Based on the central limit theorem in statistics and the methods for solving the statistical parameters of the lognormal distribution, the mean, median, mode, and the ±95% confidence intervals of the mean corresponding to the 3-Parameter Lognormal distribution are used as statistical parameters for comparison and analysis. The statistical results are presented in Table 5.

**Figure 18:**
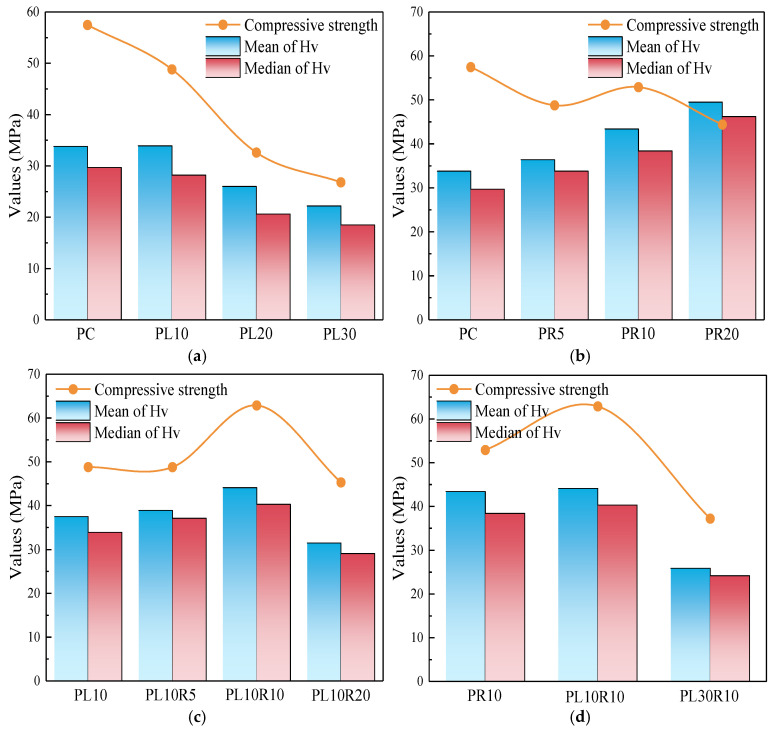
Comparison of compressive strength and statistical parameters of microhardness in (**a**) cement-LP, (**b**) cement-RHA and (**c**,**d**) cement-LP-RHA system.

The mean and median obtained from the distribution in Table 5 are close to those in Table 3. According to the central limit theorem in statistics, the limiting distribution of any independent random variable follows the normal distribution. In summary, the lognormal distribution can more accurately describe the distribution of microhardness in hardened cement stone. A comparison of the parameters in Table 5 with the compressive strength at the same age (28 d) is shown in Figure 18.

The compressive strength of cement mortar and the microhardness of cement stone show a high consistency with the varying content of LP, while the effect of RHA on compressive strength and microhardness differs. This is due to the pozzolanic reaction of RHA, which changes the Ca/Si composition of the primary hydration product (C-S-H gel), increasing the density per unit volume of C-S-H and the hardness per unit indentation. The compressive strength is influenced not only by the hardness of the hydration products but also by the pore structure of the paste, leading to some discrepancy with microhardness. As shown in Figure 18, the paste with 10% LP and 10% RHA exhibits superior mechanical property. However, at a higher LP content, there is a significant drop in the mechanical property of the composite cementitious material compared to the sample without LP. It can be seen combining with Figure 9 that at the same LP content, RHA effectively improves the mechanical property of the cementitious material and accelerates the property with the high LP content.

## 4. Conclusions

(1) Mixing RHA and LP improves the compressive strength of cement-based materials. The highest strength is achieved when both RHA and LP are at 10%, with a 9.5% increase in 28 d strength compared to the pure cement system.
(2) The addition of RHA effectively improves the slow strength development and insufficient late-stage strength of the cement-LP system, as well as the low strength of the cementitious system with high LP content. When the LP content is 20% and 30%, 10% RHA increases the 28 d strength by 44.8% and 38.8%, respectively, and with strength growth rates of 109.8% and 151.1% at 3 d–56 d.
(3) The microhardness data of cement-based materials mixed with either RHA or LP is more consistent with a lognormal distribution. RHA effectively improves the microhardness of composite hardened cement paste. The synergistic effect of the pozzolanic reaction and internal curing water release of RHA, along with the nucleation effect of LP, promotes the hydration of cement, leading to a denser interlayer structure of C-S-H. This results in cement-based materials having relatively high microhardness values.
(4) The synergistic effect of LP and RHA significantly enhances the mechanical properties of the cementitious material. Based on a comprehensive analysis of compressive strength and microhardness, the optimal solution for achieving high mechanical performance in the composite cementitious material is to use 10% substitution to cement for both LP and RHA.

## Figures and Tables

**Table 1:** Chemical compositions of the cement and RHA (wt%).

Chemicals	SiO_2_	Al_2_O_3_	Fe_2_O_3_	CaO	MgO	SO_3_	Na_2_O_eq_	Others	F-CaO	LOI
Cement	22.08	4.32	3.43	63.67	2.32	2.62	0.58	—	0.90	2.03
RHA	99.33	0.098	0.047	0.073	0.033	0.083	0.023	0.316	—	—

**Table 2:** Mix proportions of cement-LP-RHA composite pastes.

Samples	Cement (wt%)	LP (wt%)	RHA (wt%)	Superplasticizer (wt%)	Sand/Binder	Water/Binder
PC	100	0	0	0	3	0.4
PL10	90	10	0	0	3	0.4
PL20	80	20	0	0	3	0.4
PL30	70	30	0	0	3	0.4
PR5	95	0	5	0.4	3	0.4
PR10	90	0	10	0.4	3	0.4
PR15	85	0	15	0.84	3	0.4
PR20	80	0	20	1.34	3	0.4
PL10R5	85	10	5	0.4	3	0.4
PL10R10	80	10	10	0.4	3	0.4
PL10R15	75	10	15	0.84	3	0.4
PL10R20	70	10	20	1.34	3	0.4
PL20R10	70	20	10	0.4	3	0.4
PL30R10	60	30	10	0.4	3	0.4
PL30R15	55	30	15	0.84	3	0.4
PL30R5	65	30	5	0.4	3	0.4

**Table 3:** Statistical results of microhardness (MPa).

Samples	PC	PL10	PL20	PL30	PR5	PR10
Mean	36.31	37.8	31.05	24.82	36.69	43.63
Standard deviation	13.57	16.92	19.81	12.09	18.16	20.96
Maximum	126.50	130.60	148.90	95.30	163.80	136.1
Upper quartile (Q3)	43.00	44.00	34.10	28.60	41.80	52.3
Median	33.90	32.85	25.50	21.20	33.30	37.4
Lower quartile (Q1)	26.80	26.50	21.10	18.20	25.60	29.5
Minimum	17.50	17.40	15.70	11.80	10.50	19
Interquartitle range (IQR)	16.20	17.50	13.00	10.40	16.20	22.8
Range (Max-Min)	109.00	113.20	133.20	83.50	153.30	117.1
**Samples**	**PR20**	**PL10R5**	**PL10R10**	**PL10R20**	**PL30R10**	—
Mean	49.65	38.94	44.34	31.69	25.86	—
Standard deviation	19.70	13.13	18.32	11.27	7.33	—
Maximum	121.90	92.60	167.8	91.00	51.10	—
Upper quartile (Q3)	58.50	47.40	53.9	35.85	29.85	—
Median	45.15	38.15	39.55	29.20	24.75	—
Lower quartile (Q1)	37.80	29.20	32	25.00	19.95	—
Minimum	16.80	14.20	23.4	19.20	15.60	—
Interquartitle range (IQR)	20.70	18.20	21.9	10.85	9.90	—
Range (Max-Min)	105.10	78.40	144.4	71.80	35.50	—

**Table 4:** AD fitting-value of each distribution.

Samples	Normal	3-Parameter Lognormal	3-Parameter Weibull	3-Parameter Gamma
PC	4.206	0.396	1.759	0.642
PL10	6.498	0.566	2.209	1.716
PL20	12.761	1.423	3.898	3.658
PL30	9.387	1.504	3.716	2.899
PR5	4.013	1.366	3.025	1.734
PR10	5.749	0.398	1.591	1.319
PR20	4.462	1.204	2.49	1.594
PL10R5	1.83	0.537	1.282	0.64
PL10R10	5.137	0.274	1.288	0.659
PL10R20	6.9	0.699	1.928	1.268
PL30R10	2.678	0.257	0.493	0.325

**Table 5:** Statistical parameters of 3-Parameter Lognormal distribution of microhardness data (MPa).

Samples	Hv			
	Mean	Median	Mode	95% Confidence Interval
PC	36.2	33.8	29.7	(34.0, 38.6)
PL10	37.5	33.9	28.2	(34.8, 40.6)
PL20	30.3	26.0	20.6	(27.7, 33.4)
PL30	24.5	22.2	18.5	(22.8, 26.5)
PR5	36.4	33.8	29.1	(33.7, 39.3)
PR10	43.4	38.4	30.9	(39.7, 47.7)
PR20	49.5	46.2	40.2	(46.0, 53.4)
PL10R5	38.9	37.1	33.8	(36.5, 41.5)
PL10R10	44.1	40.3	34.3	(41.2, 47.5)
PL10R20	31.5	29.1	25.4	(29.8, 33.6)
PL30R10	25.9	24.2	21.5	(24.5, 27.4)

## Data Availability

The original contributions presented in the study are included in the article, further inquiries can be directed to the corresponding authors.
